# Informed consent form for platelet rich plasma injections: evidence-based and legally guide for orthopaedic surgeons

**DOI:** 10.1186/s40001-024-02019-8

**Published:** 2024-08-17

**Authors:** Madhan Jeyaraman, Satvik N. Pai, Migliorini Filippo, Naveen Jeyaraman, Ravichandran Venkatasalam, Arulkumar Nallakumarasamy, Manish Khanna, Bishnu Prasad Patro, Shilpa Sharma, Ravi Velamor Rangarajan

**Affiliations:** 1 Department of Orthopaedics, ACS Medical College and Hospital, Dr MGR Educational and Research Institute, Chennai, Tamil Nadu 600077 India; 2 Department of Biotechnology, School of Engineering and Technology, Sharda University, Greater Noida, Uttar Pradesh 201310 India; 3 Indian Stem Cell Study Group (ISCSG) Association, Lucknow, Uttar Pradesh 226010 India; 4 Department of Regenerative Medicine, Mother Cell Regenerative Centre, Tiruchirappalli, Tamil Nadu 620017 India; 5 Department of Regenerative Medicine, Orange Health Care, Chennai, Tamil Nadu 600040 India; 6 Department of Orthopaedics, PES University Institute of Medical Sciences and Research, Bengaluru, Karnataka 560083 India; 7 Department of Orthopaedic, Trauma, and Reconstructive Surgery, RWTH University Medical Centre, Pauwelsstraße 30, 52074 Aachen, Germany; 8 Department of Orthopedics and Trauma Surgery, Academic Hospital of Bolzano (SABES-ASDAA), 39100 Bolzano, Italy; 9 Department of Life Sciences, Health, and Health Professions, Link Campus University, 00165 Rome, Italy; 10 Department of Orthopaedics, Jawaharlal Institute of Postgraduate Medical Education and Research (JIPMER), Karaikal, Puducherry, 609602 India; 11 Department of Orthopaedics, All Indian Institute of Medical Sciences, Bhubaneswar, Odisha 751019 India; 12 Department of Paediatric Surgery, All India Institute of Medical Sciences, New Delhi, 110029 India

**Keywords:** Informed consent, Platelet-rich plasma, PRP, Medico-legal, Consent

## Abstract

Regarding medico-legal malpractice suits, lawyers and insurers focus on informed consent documentation. Unfortunately, there is no standard protocol for obtaining informed consent for platelet-rich plasma (PRP) injections. The objective of the present study was to create a pre-designed, evidence-based informed consent form specifically for PRP injections. The current evidence on the medico-legal implications of PRP injections was accessed, as well as informed consent in general and specifically informed consent in PRP injections. Additionally, we interviewed orthopaedic surgeons and patients who had undergone PRP injections in the past year using a semi-structured approach. A legally valid and evidence-based informed consent form for PRP injections ensures rights, encouraging open communication and transparency between the patient and surgeon. Moreover, if a lawsuit arose, informed consent would be a critical document in surgeons' defence and would withstand scrutiny from lawyers and the judiciary. An evidence-based informed consent form for PRP injections was elaborated and reviewed by a legal expert to ensure adherence to legal proprieties. The final form of the informed consent for PRP injection was administered for one year and validated at our institution.

**Supplementary Information:**

The online version contains supplementary material available at 10.1186/s40001-024-02019-8.

## Introduction

Informed consent is crucial to medical ethics and autonomy and enables patients to participate in healthcare decisions. Obtaining informed consent before a medical procedure is crucial. The medical consent ensures that the patient is fully aware of the risks, benefits, and alternatives to the procedure and can make an informed decision on his healthcare. Secondly, it helps to establish trust and rapport between the patient and the healthcare provider, leading to better outcomes and higher patient satisfaction. Furthermore, it provides legal protection for the healthcare provider in case of adverse events or complications [1–5]. Its importance has risen significantly in the past few decades. It is often the first area of interest for lawyers and insurers concerning a medicolegal malpractice suit.

Platelet-rich plasma (PRP) injections are a form of regenerative medicine that has recently gained popularity. They involve using a patient's blood drawn and processed to concentrate platelets and growth factors [6]. PRP injections are involved in the management of acute and overused musculoskeletal ailments [7, 8]. Especially in sports medicine, PRP injections are involved in the management tendinitis, ligament sprains, and muscle strains [9–12]. PRP also has applications in dermatology, urology, and other fields of medicine [13–17]. However, the absence of a consistent and established method for acquiring informed consent concerns [18]. As a result, the informed consent documents for the procedure frequently omit crucial information, which could endanger their legal validity. This underscores the importance of developing a pre-determined, evidence-supported informed consent form tailored to administer PRP injections [19]. The present study introduced and validated an informed consent form for PRP injections.

## Materials and methods

**Fig. 1:**
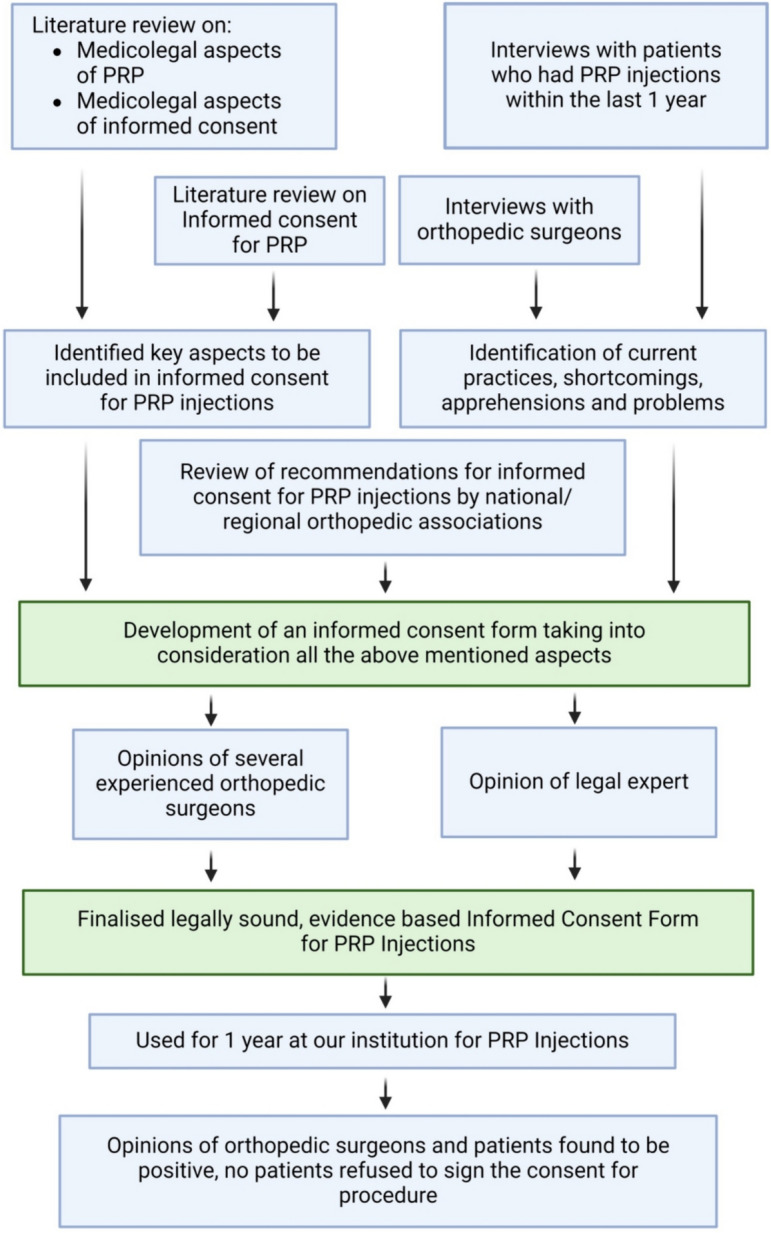
Flow diagram depicting the steps in formulating the consent form

Upon obtaining ethical clearance from our institutions [JJM Medical College, Davangere; School of Medical Sciences and Research, Sharda University, Greater Noida; Mahatma Gandhi Medical College and Research Institute, Puducherry; Faculty of Medical Sciences—Sri Lalithambigai Medical College and Hospital, Chennai; and All India Institute of Medical Sciences, Bhubaneshwar], a comprehensive review of the literature on informed consent for PRP injections was performed, including medicolegal aspects and complications. Using search terms and Boolean operators AND/OR, PubMed and Cochrane Library were accessed in 2017. We also searched for medicolegal proceedings involving PRP injection in legal courts, consumer dispute redressal forums, and state medical councils using online legal databases [IndianKanoon.com and scconline.com] and books. We documented and curated the results of our literature review. We conducted the literature review. We then wrote down the points of learning we had from the review. We then listed relevant references for what we had learned from the literature review. The points were considered while making the consent form to ensure the recommendations were incorporated into our consent form. The following keywords were used: “Informed Consent Form" AND "Platelet Rich Plasma Injections" AND "Orthopaedic Surgeons" Evidence-based" OR "Legally Sound. A semi-structured interview was conducted with orthopaedic surgeons and patients who had undergone PRP injections in the past year at our institution. We asked the orthopaedic surgeons about common practices and difficulties related to informed consent for PRP injections and patient concerns. We asked the patients about their experiences with the informed consent process, its usefulness, and any doubts not satisfactorily addressed. Based on this information, we developed an evidence-based informed consent form that was presented to experienced orthopaedic surgeons and a legal expert for further feedback. We made minor modifications based on their suggestions and prepared the final consent form. The present consent form was administered to 147 patients at our institution without any concerns raised or patients refusing to sign. The process of obtaining the informed consent was video recorded. We did not find any additional modifications needed. Both orthopaedic surgeons and patients responded positively to the consent form (Fig. 1).

### Validation of instrument

Three surgeons from the Department of Orthopaedics in Chennai, one from the Department of Forensic Medicine in Chennai, two from the Department of Community Medicine in Chennai, one expert from the Department of Orthopaedics in Delhi and Bhubaneswar, and two from the Department of Forensic Medicine in Patna and the Department of Community Medicine in Mumbai were included in the present study. These assessors received all consents in a printed version and objectives, blueprints, and criteria rating scales. After validating the content, all the tools were returned.

### Baseline proforma of the study participants

Most items in the informed consent proforma were agreed upon unanimously. The proforma consisted of 19 items for study participants, but some modifications were made based on the suggestions of the validators. After expert consultation, two items had less than 60% agreement and were subsequently removed. Consequently, the informed consent form for the present study had 17 items.

To assess the content validity rate (CVR), the questionnaire was distributed to 6 experts with specialisations related to the study's field. The questionnaire included answers based on a three-point Likert scale that classified items as necessary, helpful but not necessary, or not necessary. Afterwards, the CVR of the questionnaire was evaluated, and items with scores above 0.95 were considered necessary and appropriate, following the Lawsche table. Items with lower scores, those unable to measure the desired concept or those with little connection to the issue were excluded based on expert feedback and respondent comments.

To examine the indexes of "relevance," "clarity," "simplicity," and "ambiguity," experts were asked to provide their opinions and suggestions regarding the items that should be included in the questionnaire. A separate content validity index (CVI) was calculated for each item and scale. Therefore, we calculated the scale-content validity index S-CVI/Average for the six constructs (relevance, clarity, simplicity, ambiguity) to be 0.94, using the experts' responses and suggestions [20].

## Results

A standardised, evidence-based, and legally sound informed consent form for PRP injections is given in Form A.

## Discussion

According to the literature, informed consent is crucial in defending against malpractice claims; however, studies suggest that the informed consent process among orthopaedic surgeons is incomplete and needs improvement [21]. This incomplete informed consent can leave orthopaedic surgeons at risk for malpractice claims, which can be used against them in court. The use of generic forms for different procedures is insufficient as they do not provide adequate documentation of complications that could arise from the procedure. Instead, procedure-specific consent forms are recommended for orthopaedic procedures [22]. Documenting the patient's diagnosis and discussing all alternative treatment options with them before surgery is important, as this is often overlooked in informed consent forms [23]. It is also essential to explain the procedure to the patient in a way that is easy to understand and for the surgeon to discuss the goals of the surgery to manage expectations. Moreover, patients should be informed that the procedure may not resolve their symptoms completely [24].

PRP injections are generally regarded as a very safe procedure, with major adverse effects being rare occurrences [25]. However, it is important to inform patients of the possible common complications and any rarer, serious complications that may arise. There is often a lack of uniformity among orthopaedic surgeons regarding which complications to include in informed consent, so it is advisable to follow guidelines set forth by a national or regional association of orthopaedic surgeons. Unfortunately, such recommendations are lacking in many countries, which has resulted in an absence of standard practice and uniformity. The consent form lists all relevant complications based on available literature and common litigation causes in PRP injection cases [26–34]. Level 1 evidence is available for using PRP in knee osteoarthritis [35], tennis elbow [36], plantar fasciitis [37], patellar tendinopathy [38], achilles tendinopathy [39], adhesive capsulitis [40], degenerative disc disease [41], rotator cuff tears [42], and ACL repair/reconstruction/augmentation [43]. More robust evidence is needed for using PRP in delayed and non-union fractures, meniscus augmentation, bursitis, tenosynovitis, ligament sprain and tears, muscle injuries, and avascular necrosis of the femoral head. Additionally, it is often overlooked that patients should be counselled regarding the possible need for multiple injections and supplementation with other forms of treatment, as this is important information for the patient to be aware of following the procedure [44, 45]. Another unique aspect of obtaining informed consent for PRP injections is that the use of PRP has only become popular within the last two decades. While substantial evidence exists for its effectiveness in managing certain conditions, it is still being explored for newer indications [46]. Therefore, we have included a clause in our consent form to acknowledge this fact. This clause has been introduced to counter legal arguments that using PRP for a particular indication is not yet an established standard practice. Users can remove the clause if the PRP injection is used for an indication with undisputable, widely accepted evidence of utility for a particular condition. Finally, it is always recommended that prior consent be obtained for photography or recording of the surgery for educational purposes or publication in scientific journals as a part of research ethics [47].

Obtaining informed consent using this consent form for patients who do not speak English can pose a legal problem. To ensure the validity of consent for such patients, we recommend documenting the language to which it was translated, the translator's details, and their signature. Anyone who can read English and translate it to the patient's language can be the translator. The patient's and doctor's signatures are necessary, and having a witness sign the form is recommended but not legally required. Currently, the consent form is only available in English, and cross-cultural validations should be done to make it available in other languages.

This consent form is based on medical evidence and legal review, but its objective assessment in terms of law is hardly possible. A legal trial analysing and discussing this consent form can make it more reliable, but it will only be valid for that specific case. That being said, it is always better to be prepared and comply with the law, and we believe that this consent form will be useful in that regard. We must also remember that the laws of each country vary from other countries. Legal principles and legal judgements unique to each country impact the laws on informed consent. The consent form we have suggested is in keeping with the informed consent requirements in India but can be a very useful blueprint for formulating such a form for other countries. It may require minor modifications specific to each country.

## Conclusion

A legally valid and evidence-based informed consent form for PRP injections can prove advantageous for orthopaedic surgeons and their patients. This type of consent would protect the patient's rights and encourage open communication and transparency between the patient and surgeon. Moreover, if a lawsuit arose, it would serve as a critical document in the surgeon's defence and withstand scrutiny from lawyers and the judiciary.

### Supplementary Information

Suppleementary Material 1

## Data Availability

All data and materials are available on reasonable request to Prof Madhan Jeyaraman (madhanjeyaraman@gmail.com).

## Abbreviations

PRP: Platelet-rich plasma
CVR: Content validity rate
CVI: Content validity index
