# Supplementary material for: Informed consent form for platelet rich plasma injections: evidence-based and legally guide for orthopaedic surgeons
Source: Eur J Med Res. 2024 Aug 17;29:422. doi: 10.1186/s40001-024-02019-8 (PMC11330123; doi:10.1186/s40001-024-02019-8)
Supplement: Supplementary file 1 — Suppleementary Material 1 [file 40001_2024_2019_MOESM1_ESM.docx]

**Form A:** A standardised informed consent form for PRP injections which is evidence-based and legally sound.

1. I, _________________________ (Name of the patient), aged ______ years, with hospital ID/ID No. __________________ hereby authorise the performance of the procedure of INJECTION OF AUTOLOGOUS PLATELET RICH PLASMA (PRP) at/in the _______________________________
2. I have been explained that I/the patient have been diagnosed to have _____________________________________________ .
3. I have been informed regarding the other treatment options for the condition including ____________________________________________________________________.
4. I have been explained briefly regarding the steps of the procedure, including the drawing of blood, processing and injection of PRP at the desired site.
5. I also understand that the procedure does not guarantee complete resolution of symptoms nor does it necessarily cure the existing medical problem. I have also been informed that it may be required to be supplemented with other forms of treatment as well.
6. I authorise Dr. _____________________­­­­____ and such associates and assistants as may be selected by him/her to perform the procedure upon myself/the patient.
7. I have been explained that while the procedure is generally safe in most cases, there exists the possibility of complications occurring during/following the procedure. The possible complications that can occur include, but are not restricted to, pain at the injection site, infection, bleeding, nerve damage, temporary lymph node enlargement, and serum sickness syndrome.
8. I have been explained about the possible use of a local anaesthetic to decrease the pain of the injection, and also been told about its potential risks.
9. I also understand that multiple sessions of PRP injection may be required to attain the desired result. I have been informed that the effects of PRP injections are not necessarily permanent.
10. I have been informed the PRP being used will be ____________________ [autologous (from my own blood) or allogenic (from the blood of another individual)]. If allogenic PRP is to be used, I have accordingly been informed about possible additional risks like allergic reactions, and spread infections such as HIV and Hepatitis.
11. I have been informed that while there is widespread evidence of the efficacy of PRP injections, it remains an evolving modality of treatment requiring further large-scale studies and evidence for particular indications.
12. I have been explained about the estimated approximate cost of the procedure.
13. I consent to the photography/recording/viewing of the procedure for the purpose of advancing medical education, or its publication in scientific journals/presentations, provided my/the patient’s identity is not revealed in any of the texts/images/videos.
14. I have fully read this consent form and comprehend all the above mentioned points.

OR

This consent has been translated to me to the language of _______________ that I can understand by _____________________ with designation/address _____________________________________________

Signed _________________

1. I hereby give my consent for the procedure after having fully understood all the above aspects.

Name: ___________________________

Signature: _________________________

Date and Time: ___________________________

If consent is being obtained from someone on behalf of the patient, the reason for inability of patient to sign the consent form _____________________, and relation of individual to the patient___________________________.

1. Declaration by doctor:

All the above points have been explained in detail to the patient/patient representative prior to surgery.

Name: ________________________

Designation: ______________________

Signature: _______________________

Date and Time: ______________________

1. Declaration by witness (Optional):

I have been present and witnessed the above said doctor/translator explain the above-mentioned points to the patient/patient representative.

Name: _____________________

Designation/Address: ___________________________

Signature: _____________________

Date and Time: ________________________
